# Heavy hematuria requiring cystectomy in a patient with hemophilia A: a case report and literature review

**DOI:** 10.1186/s12894-015-0076-8

**Published:** 2015-08-13

**Authors:** Satoshi Washino, Masaru Hirai, Yutaka Kobayashi, Kimitoshi Saito, Tomoaki Miyagawa

**Affiliations:** The Department of Urology, Saitama Medical Center Jichi Medical University, 1-847, Amanuma-cho, Omiya-ku, Saitama city, Saitama Japan

## Abstract

**Background:**

Hemophilia A is an X-linked recessive disorder caused by a deficiency in factor VIII. Hemophilia A affects 1 in 5,000–10,000 males. Hematuria is frequent in hemophilia. Hematuria in hemophilia is generally considered benign and manageable with conservative therapy; however, severe hematuria requiring surgical therapy has rarely been reported.

**Case presentation:**

A 60-year-old male with hemophilia A presented with persistent gross hematuria of unknown cause. He was treated with recombinant factor VIII products, followed by several conservative therapies as follows: clot evacuation by vesicoclysis, continuous bladder irrigation with normal saline, and intravesical instillation of aluminum hydroxide/magnesium hydroxide (Maalox); however, these failed to resolve the hemorrhaging. The patient was offered and consented to cystectomy with an ileal conduit. Intraoperative clotting was normal with the infusion of adequate recombinant factor VIII products and transfusion of fresh-frozen plasma, and the procedure was performed safely. After surgery, the patient had blood in his stool several times. No bleeding site was demonstrated in the colon by colonoscopy and ^99m^Technetium-human serum albumin-diethylenetriaminepenta-acetic acid scintigraphy demonstrated that the extravasation of radioactive isotope was detected at the anal side of terminal ileum but not at the oral side. These findings were suspected to be bleeding from the ileoileal anastomosis. However, the bleeding was managed with recombinant factor VIII products.

**Conclusions:**

Cystectomy in hemophilia may be safe, if monitored appropriately. Urinary diversion using the intestine may be avoided because anastomotic hemorrhage may become a problem.

## Background

Hemophilia A and B are X-linked recessive disorders caused by deficiencies in factors VIII and IX, respectively. Hemophilia A affects 1 in 5,000–10,000 males, whereas hemophilia B affects 1 in 25,000–30,000 males [1]. Hematuria is a frequent manifestation of hemophilia. Historically, hematuria in hemophilia has generally been considered benign and manageable with conservative therapy [1]. However, severe hematuria in hemophilia has rarely been reported [2, 3]. Here, we present a case of severe hematuria requiring surgical therapy. This is the first case report to date of cystectomy with an ileal conduit urinary diversion in a patient with hemophilia A.

## Case presentation

A 60-year-old male with a mild factor VIII deficiency presented to the hematology clinic at our hospital with a 1-week history of asymptomatic gross hematuria. He had suffered a hemorrhagic gastric ulcer at the age of 48 and was diagnosed with mild hemophilia A (his factor VIII levels were 6 % of normal) at that time. He had suffered a cerebral hemorrhage at the age of 59. For several years beginning at the age of 50, the patient had experienced mild hematuria, and he had experienced one episode of intramuscular and subcutaneous hemorrhage; both conditions were managed with recombinant factor VIII products.

**Fig. 1:**
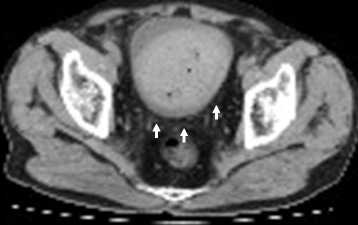
Computed tomography demonstrated that the bladder was filled with a blood clot (arrow)

A physical examination revealed no abnormal signs. Laboratory tests revealed that the patient’s activated partial thromboplastin time (aPTT) was prolonged to 74.6 s (normal range, 28.5–40.9 s), but his prothrombin time-international normalized ratio, platelet count, serum creatinine level, and prostate-specific antigen level were unremarkable. The patient’s urinalysis results were normal, except for the gross hematuria, and urine cytology revealed no cancer cells. The patient was treated with a third-generation recombinant factor VIII product (Advate). However, he had persistent hematuria, followed by clot retention. Thus, he was referred to the Department of Urology. Computed tomography demonstrated that his bladder was filled with a blood clot (Fig. 1), but his prostate and upper urinary tract were apparently normal.

He was admitted to our hospital and received the following therapy: clot evacuation by vesicoclysis, continuous bladder irrigation with normal saline, and intravesical instillation of aluminum hydroxide/magnesium hydroxide (Maalox) concurrent with the administration of Advate, which failed to resolve the hemorrhage. Consequently, the patient had repeated transfusions of packed red cells. Although the patient also underwent transurethral coagulation of the bladder mucosa under anesthesia, the bleeding presented with oozing throughout the mucosa and was not controlled. Subsequently, the patient developed pyelonephritis in his left kidney with a severe reduction (<50 mL) in his bladder capacity. Conservative management for 2 months failed to resolve the patient’s symptoms, so he was offered a cystectomy with ileal conduit and consented to it after a detailed discussion.

The quality of intraoperative clotting seemed to be normal with the use of sufficient Advate to raise the levels of factor VIII to 100 % (3000 U, IV bolus) and the transfusion of 3 U of fresh-frozen plasma. His aPTT was 54 s during surgery. The cystectomy was performed safely without severe bleeding events. A 15-cm length of ileal segment at 15 cm from the ileocecal valve was used for the ileal conduit. The technique of side-to-side stapled anastomosis was used for the ileoileal anastomosis, and the Bricker anastomosis technique was used for ureteroileal anastomosis. The total operating time was 220 min and the estimated blood loss was 800 mL.

The resected specimen revealed multiple erosions and ulcers in the bladder mucosa, and sclerosis of the bladder wall. Histological examinations demonstrated inflammatory cell infiltration and fibrous changes in the bladder wall without malignant figures. The cause of the hematuria was unclear.

Postoperatively, Advate (3000 U, q12 h) was administered to maintain 100 % levels of factor VIII for 2 days with no bleeding complications. After removal of the ureteral catheters on postoperative day 13, the patient had urinary leakage from the ureteroileal anastomosis, which induced a pelvic abscess followed by septic shock and acute respiratory distress syndrome. Blood cultures were positive for *Candida tropicalis*. Several antibiotics and surgical drainage of the abscess were needed.

**Fig. 2:**
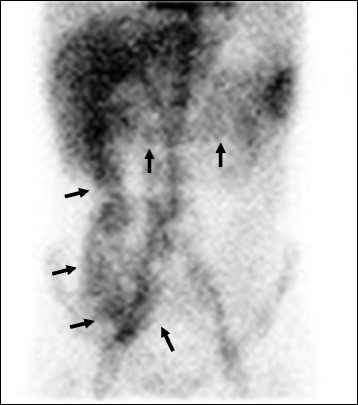
^99m^Technetium-human serum albumin-diethylenetriaminepenta-acetic acid scintigraphy demonstrated that the extravasation of radioactive isotope was detected at terminal ileum, cecum, ascending and transverse colon (arrow), but not at the oral side of terminal ileum

At 4 months after the cystectomy, the patient had blood in his stool requiring a transfusion of packed red cells. No bleeding site was demonstrated in the colon by colonoscopy and ^99m^Technetium-human serum albumin-diethylenetriaminepenta-acetic acid scintigraphy demonstrated that the extravasation of radioactive isotope was detected at terminal ileum, cecum, ascending and transverse colon but not at the oral side of terminal ileum (Fig. 2). These findings were suspected to be bleeding from the ileoileal anastomosis. The patient was given Advate (2000–4000 U) three times per week for 6 months, and the blood in his stool resolved. His condition recovered gradually and he was discharged at 8 months after admission.

After discharge, he had mild gastrointestinal bleeding and mild hematuria several times a year; however, this was controlled by Advate. The patient had been doing well, other than the bleeding tendency, until he suffered a malignant lymphoma at the age of 66 and was transferred to another clinic for treatment.

## Discussion

Hemophilia A and B are rare X-linked recessive disorders caused by deficiencies in factors VIII and IX, respectively. They exhibit a range of clinical severity that correlates well with assayed factor VIII and IX levels. Severe disease is defined as < 1 % factor activity, whereas 1–5 % and > 5 % of normal are deemed moderate and mild disease, respectively [4]. Hematuria is a frequent manifestation of hemophilia. In two studies of hemophiliacs, 66 % had a history of hematuria [5, 6]. The etiology of hematuria is broad, ranging from benign causes to potentially life-threatening malignancies, including bladder cancer, renal cell cancer, and prostate cancer [7]. However, the etiology of hematuria in hemophilia is often unclear, and it may be attributable to the underlying coagulation deficiency [1]. Historically, hematuria in hemophiliacs is generally considered benign in nature and usually responds to conservative therapies, including hydration, bed rest, and infusions of factor concentrate [1]. However, severe hematuria in classic or acquired hemophilia has rarely been reported [2, 3]. In our case, severe hematuria occurred without any obvious cause except the hemophilia, and persisted despite several conservative therapies. After failure of several conservative therapies we next chose a surgical treatment, cystectomy, because he had not only severe hematuria but also pyelonephritis with a severe reduction in his bladder capacity although selective embolization of any major bleeding vessels prior to cystectomy may also be one of treatment options. Histological analyses, which indicated non-specific inflammatory cell infiltration in the bladder, also failed to demonstrate the cause of the hemorrhage.

Performing a surgical procedure in hemophiliac patients is a demanding challenge for the surgeon and hematologist. Various open abdominal operations, including appendectomy, cholecystectomy, splenectomy, and gastric/intestinal procedures, in patients with hemophilia of varying degrees of severity have been reported [8]. In the urology field, successful prostatectomies for prostate cancer in hemophiliac patients have been reported [9, 10]. However, cystectomy in a hemophiliac patient has not been reported before. In our case, with adequate intraoperative factor replacement therapy and the transfusion of fresh-frozen plasma, the quality of intraoperative clotting seemed to be normal and the cystectomy was performed safely.

Our patient experienced urinary leakage from the ureteroileal anastomosis after removal of the ureteral stent, leading to abscess formation and sepsis. The time to stent removal on postoperative day 13 was supposed to be sufficient. The rate of urinary leakage from an anastomosis has been reported to be ~2 % with the routine use of soft plastic stents placed across the ureteroenteric anastomosis [11]. However, it has been reported that wound healing is impaired in hemophilia but that normal healing can be obtained with adequate hemostatic replacement therapy for 7 days [12, 13]. It is possible that tissue healing at an anastomosis is also impaired in hemophilia. Adequate factor VIII concentrate for an extended period of time and the delay of stent removal at an anastomotic site may be required in hemophiliac patients. Stentogram before removal of ureteric stent after ileal conduit construction may also be required to detect leakage of urine at the ureteroenteric anastomosis in hemophiliac patients although routine postoperative stentogram is not recommended in patients with a normal postoperative course [14].

Our patient also had intestinal bleeding, probably from the ileoileal anastomosis. Generally, anastomotic hemorrhage is considered a rare entity. To our knowledge, there is no previous report on an ileoileal anastomotic hemorrhage. The incidence of colorectal anastomotic hemorrhage is reported to be as high as 5 % [15]. There is one report of a severe anastomotic hemorrhage in a hemophiliac patient after total gastrectomy with a Roux-en-Y reconstruction [16]. The intestinal bleeding in our case was likely attributable to his bleeding tendency. Because anastomotic bleeding may be serious in hemophilia, urinary diversion using the intestine should be avoided if possible.

## Conclusion

To our knowledge, this is the first case report of cystectomy in a patient with hemophilia A. In our case, the procedure was performed safely with normal intraoperative clotting, but ureteroileal anastomotic leakage and intestinal bleeding were problematic.

## Consent

Written informed consent was obtained from the patient for publication of this case report and any accompanying images. A copy of the written consent is available for review by the Editor of this journal.

## Abbreviation

aPTT: Activated partial thromboplastin time
